# MRI Quantification of Liver Fibrosis Using Diamagnetic Susceptibility: An Ex Vivo Validation Study

**DOI:** 10.3390/tomography12040046

**Published:** 2026-03-31

**Authors:** Chao Li, Jinwei Zhang, Alexey V. Dimov, Anne K. Koehne de González, Martin R. Prince, Jiahao Li, Dominick Romano, Pascal Spincemaille, Thanh D. Nguyen, Gary M. Brittenham, Yi Wang

**Affiliations:** 1 Department of Radiology, Weill Cornell Medicine, New York, NY 10065, USA; 2 Applied and Engineering Physics, Cornell University, Ithaca, NY 14853, USA; 3 Meinig School of Biomedical Engineering, Cornell University, Ithaca, NY 14853, USA; 4 Department of Pathology, Columbia University, New York, NY 10032, USA; 5 Department of Pediatrics, Columbia University, New York, NY 10032, USA

**Keywords:** liver fibrosis, quantitative susceptibility mapping, susceptibility source separation

## Abstract

Background/Objectives: Liver fibrosis, if left untreated, can lead to cirrhosis and cancer. The current standard liver biopsy for fibrosis staging is invasive and prone to risks of complication. The objective of this study was to develop a new noninvasive method to quantify fibrosis using diamagnetic susceptibility sources generated from multi-echo gradient echo (mGRE) data with both magnitude decay R2* modeling and phase QSM modeling. Methods: mGRE data of ex vivo liver explants was processed with fat–water separation and then susceptibility source separation. Negative susceptibility was used to measure diamagnetic fibrosis. In 20 formalin-fixed liver explant sections, negative susceptibility maps were compared with other MRI parameters against pathology for fibrosis staging. Results: The correlation between the negative susceptibility sources and the fibrosis stages was evaluated with Spearman coefficients. Negative susceptibility differentiated (i) no or mild fibrosis (stages F0 to F1) from moderate-to-advanced fibrosis (stages F2 to F3; *p* = 0.0025), (ii) stages F2 to F3 from cirrhosis (stage F4; *p* = 0.021), and (iii) no-to-moderate fibrosis (stages F0 to F2) from advanced fibrosis or cirrhosis (stages F3 to F4) with a sensitivity of 90%, a specificity of 90%, and a 0.88 Receiver Operating Characteristic Area Under the Curve (AUC) (*p* = 0.0017). Conclusions: For staging fibrosis, negative susceptibility was superior to other MRI parameters, including R2*, QSM, and PDFF. Negative susceptibility sources were positively correlated with the fibrosis stage (r = 0.60). Negative susceptibility could be valuable for MRI staging in liver fibrosis.

## Simple Summary

Liver fibrosis is a serious condition that can progress to cirrhosis and liver cancer, but the current standard test, liver biopsy, is invasive and risky. This study introduces a new magnetic resonance imaging method that measures tissue properties related to scar formation in the liver without surgery. Using donated liver samples, we show that this technique accurately distinguishes mild, moderate, and advanced fibrosis and performs better than several existing MRI measurements. This approach could help radiologists assess liver disease more safely, monitor treatment response, and support future studies toward noninvasive clinical diagnosis.

## 1. Introduction

Fibrosis is the best predictor of morbidity and mortality in chronic liver disease [1,2]. Liver biopsy, the current reference method for the diagnosis and staging of hepatic fibrosis, is invasive; cannot be used for frequent, repeated monitoring; and is limited in spatial sampling and complication risks and by interobserver variability. Consequently, alternative methods that avoid biopsy are needed for patient care and clinical research [3]. The recent FDA approval of the selective thyroid hormone receptor β agonist resmetirom as the first drug for the treatment of adults with noncirrhotic, nonalcoholic steatohepatitis (NASH, MASH, or MASLD) can be expected to increase the demand for noninvasive means to evaluate hepatic fibrosis [4,5].

Noninvasive quantitative MRI methods for the diagnosis and staging of fibrosis offer unique advantages in characterizing local intrinsic liver tissue properties. Ultrasound-based transient elastography (TE, also called ultrasonography-based transient elastography, UTE) [6] and magnetic resonance elastography (MRE) [7] quantify shear-wave propagation and show moderate diagnostic performance [8,9]. MRI with diffusion weighting (DWI) consistently shows lower apparent diffusion coefficients in fibrotic livers than in healthy controls [10]. Other MR biomarkers, such as T1 and T2 relaxometry, also appear promising for grading disease severity [11]. However, each modality has practical constraints: TE/UTE performance falls in patients with obesity due to reduced penetration and often requires additional hardware [12]. MRE requires expensive equipment and specialized expertise. T2 relaxometry suffers from high inter-study variability [13].

Quantitative susceptibility mapping (QSM) derived from complex multi-echo gradient echo (mGRE) data [14] also enables the specific quantitative study of major liver susceptibility sources, diamagnetic fibrosis and paramagnetic iron. Recently, decay rate R2* from mGRE magnitude data and QSM from mGRE phase data were combined via regression to distinguish between the different stages of fibrosis in ex vivo liver explants [15]. However, R2* and QSM can be combined via optimization to generate susceptibility source separation (R2*QSM) [16,17], including negative susceptibility mapping specific to diamagnetic fibrosis.

Here, we describe liver fibrosis quantification using negative susceptibility determined from mGRE data using susceptibility source separation [18]. Referencing it to the semiquantitative histological staging of fibrosis, we investigated the negative susceptibility and the individual parameters R2*, PDFF and QSM to differentiate between samples with no, mild or moderate fibrosis and those with advanced fibrosis or cirrhosis.

## 2. Materials and Methods

### 2.1. Liver Samples

This study was approved by the Institutional Review Board. Between January 2021 and November 2023, liver explant samples were collected by a liver pathologist. Sections of the livers of ~7 × 5 × 1.5 cm^3^ size and ~40 g weight were selected, preserved in formalin, and subsequently imaged with MRI.

### 2.2. MRI Acquisition

Liver samples preserved in formalin were placed in a cylindrical agarose mold covered with a water balloon. MRI was performed on a 3T scanner (GE Healthcare, Waukesha, WI, USA). Imaging sequence was 3D mGRE with 8 echoes, flip angle = 15, TE1 = 2.6 ms, ΔTE = 2.7 ms, TR = 24.43 ms, reconstructed voxel size = 0.88 × 0.88 × 1 mm^3^, bandwidth = 390 Hz/pixel, reconstructed matrix = 256 × 256 × 74 – 128.

### 2.3. MRI Analysis—R2, R2*, and Fat–Water Separation

MRI analysis was conducted using MATLAB (version R2023b; MathWorks, Natick, MA, USA). R2* values were derived from the magnitudes of the mGRE using the ARLO method [19]. An initial estimation of the field *f* was produced from the complex mGRE signals S(t) with N echoes, achieved through concurrent phase unwrapping and chemical shift elimination (SPURS) [20]. The IDEAL algorithm [21] was applied to create maps of water (W), fat (F), and field (f) mGRE data from a single-peak fat model:

(1) $$E\left(W,F,f\right)={\mathrm{argmin}}_{W,F,f}\sum_{j=1}^{N}\;{∥S\left(t_{j}\right)-e^{-R_{2}^{*}t_{j}}e^{-i2\pi ft_{j}}\left(W+Fe^{-i2\pi \nu \cdot t_{j}}\right)∥}_{2}^{2}\;,$$

The background field was removed using the Projection onto Dipole Fields (PDF) technique [22]. The local field map was then used to produce a susceptibility map (*χ*) through the Morphology Enabled Dipole Inversion (MEDI) algorithm [23,24,25]. The susceptibility measurements were referenced to the mean susceptibility value of the water balloon [24]. Images representing the Proton Density Fat Fraction (PDFF) were generated from the computed fat and water images *as* F/(W+F) [26].

To exclude the background agarose gel and air, liver specimen volumes were automatically created by combining threshold-based segmentation with manual segmentation. This involved generating a liver binary mask $M_{L}$ using an R2* ≥ 15 s^−1^ threshold [15]. The mask was then manually eroded around large blood vessels, and any air bubbles were also excluded to prevent inaccuracies in fibrosis detection. Finally, the average values of all MRI parameters were calculated within the mask.

### 2.4. MRI Analysis—Susceptibility Source Separation

QSM χ susceptibility at a voxel was considered to be the sum of positive source $\chi^{+}$ and negative source $\chi^{-}$:

(2) $$\chi =\;\left|\chi^{+}\right|-\left|\chi^{-}\right|,$$

These positive and negative sources were assumed to contribute additively to the static dephasing rate $R_{2}^{*}$ [18]:

(3) $$R_{2}^{*}\approx \;r^{+}\left|\chi^{+}\right|+r^{-}\left|\chi^{-}\right|,$$

The dephasing constants $r^{+}=r^{-}$ were assumed to be the same and may vary with tissues.

The positive and negative susceptibility maps were calculated by solving the optimization problem with regularization [17,18,27], which can be formulated as:

(4) $$\begin{array}{c} \chi^{+*},\chi^{-*}=arg\underset{\chi^{+},\chi^{-}}{min}\;{∥w_{1}\left(R_{2}^{*}-r\left(\left|\chi^{+}\right|+\left|\chi^{-}\right|\right)\right)∥}_{2}^{2}+{∥w_{2}\left(f-d*\left(\chi^{+}+\chi^{-}\right)\right)∥}_{2}^{2}\\ \;\;\;\;\;\;\;\;\;\;\;\;\;\;\;\;\;\;\;\;\;\;\;\;\;\;\;\;\;+2\lambda_{1}{\left|M_{E\;}\nabla \left(\chi^{+}+\chi^{-}\right)\right|}_{1}+\lambda_{1}{\left|M_{L}\nabla \chi^{+}\right|}_{1}+\lambda_{1}{\left|M_{L}\nabla \chi^{-}\right|}_{1}\\ \;\;\;\;\;\;\;\;\;\;\;\;\;\;\;\;\;\;\;\;+\lambda_{2}{∥M_{b\;}\left(\chi^{+}-\bar{\chi_{b}^{+}}\;\right)∥}_{2}^{2}+\lambda_{2}{∥M_{b\;}\left(\chi^{-}-\;\bar{\chi_{b}^{-}}\right)∥}_{2}^{2}\;, \end{array}$$

where f is the local field as in Equation (1), $\lambda_{i}$ are regularization parameters, ∇ is a gradient operator, $M_{E}$ is a binary edge mask derived from the magnitude image, $M_{L}$ is a binary edge mask derived from $R_{2}^{*}$ $,M_{b}$ is a binary mask of the water balloon, and $\bar{\chi_{b}^{+}}$ and $\bar{\chi_{b}^{-}}$ are $\chi^{+}$ and $\chi^{-}$ averaged over, respectively. $w_{1}$ and $w_{2}$ are data weighting terms. Susceptibility values violating $\chi^{+}>0$ and $\chi^{-}<0$ were reset to zero.

### 2.5. Relaxometry Estimation—Learning from Histology with Leave-One-Out Cross-Validation

The dephasing constant may be calculated for the brain assuming only a positive susceptibility source in deep gray matter regions [18]. This assumption of regions with a single susceptibility source is problematic in general and particularly for the liver tissue where there is a heterogeneous mixture of diamagnetic fibrosis and paramagnetic iron and fat. In this work, we estimated the dephasing constant r such that the obtained negative susceptibility best differentiated various fibrosis groups. This was achieved by minimizing the loss of the log-likelihood for the logistic models F0–1 vs. F2–3, F2–3 vs. F4 and F0–1 vs. F4:

(5) $$\log Loss=-\sum_{j=1}^{3}\sum_{i=1}^{N_{j}}\;\left[y_{i}^{j}log\left(p_{i}^{j}\right)+\left(1-y_{i}^{j}\right)log\left(1-p_{i}^{j}\right)\right],$$

Here j=1 stands for the logistic model differentiating F0–1 vs. F2–3, j=2 stands for the model differentiating F2–3 vs. F4, and j = 3 for F0–1 vs. F4. When a decay constant r is given, an average negative susceptibility can be solved rapidly using Equations (2) and (3). This value for each liver sample was used as the input of the logistic models to predict the labels of the sample. $y_{i}^{j}$ is the label for sample i in model j (for example, if $y_{i}^{j}=1$, the model only contained data with F0–1 and F2–3), and $N_{j}$ is the total number of samples for this model. $p_{i}^{j}$ is the prediction of sample by model j given the average negative susceptibility for sample i. We examined the values for D from 50 Hz/ppm to 300 Hz/ppm with a step of 1 Hz/ppm. After obtaining the optimal value for r, we generated the final susceptibility map using Equation (4).

To avoid the “double dipping” problem and given the small dataset size, a leave-one-out cross-validation approach was employed for subject-specific models, where each liver sample was withheld while the remaining N-1 patients were used for training to get the optimal r using Equation (5). Therefore, the susceptibility in later analysis for each liver sample was based on the r value that was independently determined from other samples.

In the results, we will compare source separation with optimized decay constant r for the liver obtained using Equation (5) and with default r = 262 Hz/ppm reported in the brain source separation literature [16,17].

### 2.6. Histopathological Analysis

A small section of each scanned liver sample was collected for histopathological analysis for each case. Hematoxylin and eosin (H&E), Masson’s trichrome, and Prussian blue stains were performed for histology, fibrosis, and iron evaluation, respectively. Fibrosis appeared as blue fibers on liver tissue samples stained with Masson’s trichrome examined under a microscope. Iron appeared as blue microscopic granules within cells on Prussian blue staining. A liver pathologist evaluated the sections and assigned scores for fibrosis, iron, and fat using standard clinical scoring systems [28,29,30]. The fibrotic stages evaluated from the small sampled sections were consistent with the histological evaluations of the original explants from the clinical side across all cases, indicating representative sampling and reliable correlation between MRI and pathology.

### 2.7. Statistical Analysis

Differences in average R2*, PDFF, $\chi ,\;|\chi^{+}|$ and $\left|\chi^{-}\right|$ between the subgroups F0–1 (non-fibrotic, mild fibrotic), F2–3 (medium-stage fibrotic) and F4 (cirrhotic) and between the subgroups F0–2 (non-fibrotic, mild fibrotic and significant-stage fibrotic) and F3–4 (advanced-stage fibrotic) liver samples were evaluated using Mann–Whitney U tests.

Since NASH patients with >F2 are the target population for pharmacological treatment and are most likely to benefit from antifibrotic drugs [31], the diagnostic accuracy of individual parameters for fibrosis detection between subgroups of liver samples with stages F0–2 and F3–4 was evaluated through Receiver Operating Characteristic (ROC) curve analysis. The optimal diagnostic threshold was determined using the Youden index, from which sensitivity and specificity values were calculated at this threshold.

We assessed the relationship between negative susceptibility sources and the histology-derived fibrosis stage with Spearman’s rank correlation. A Spearman coefficient close to +1 indicates a strong positive association, a value near −1 signifies a strong negative association, and one near 0 suggests no association. This rank-based test is appropriate because it only requires that each variable can be ranked.

Finally, we evaluated the ability of $\left|\chi^{+}\right|$ to differentiate iron content among the no-iron group, grade 0–1 iron (I0–1, including samples with grade 0, grade 0–1 and grade 1 iron as shown in Table 1), and grade 2–3 iron (I2–3, including samples with grade 2, grade 2–3 and grade 3 iron) using Mann–Whitney U tests. Although the primary goal of the study is to develop a noninvasive diagnostic tool for liver fibrosis, this analysis provides additional support for the method.

## 3. Results

### 3.1. Demographics and Histopathological Characteristics

A total of 22 patient samples underwent scanning, with 20 of these being subjected to analysis. Two samples were excluded from the analysis due to the failure of water–fat separation, attributed to extremely high R2* values caused by iron overload. These excluded samples only displayed a discernible liver signal in the initial echo of mGRE images.

Table 1 summarizes the demographics and histopathological characteristics across the entire cohort and the different fibrosis groups (F0–1 vs. F2–3 vs. F4). Among the 20 samples analyzed, eight displayed liver cirrhosis (stage 4), two indicated stage 3 fibrosis, five were identified with stage 2 fibrosis, and two with stage 1 fibrosis. The other three samples did not present any signs of fibrosis, leading to a classification of five samples as either non-significant fibrosis or non-fibrotic (F0–1, combining stages 0 and 1) and seven as intermediate-stage fibrosis (F2–3, combining stages 2 and 3). Iron deposition, noted either in hepatocytes or Kupffer cells, was observed in 10 samples, with seven belonging to the F4 group, two to the F2–3 group, and one to the F0–1 group, indicating a noticeable trend towards a higher prevalence of iron deposition among the cirrhotic samples. Steatosis exceeding 5% was observed in six samples, including three from the F4 group, one from the F2–3 group, and two from the F0–1 group. Notably, four samples within the cirrhotic group exhibited minimal steatosis (less than 5%).

### 3.2. $\chi^{-}$, $\chi^{+}$, R2*, PDFF, and QSM Measurements

**Figure 1:**
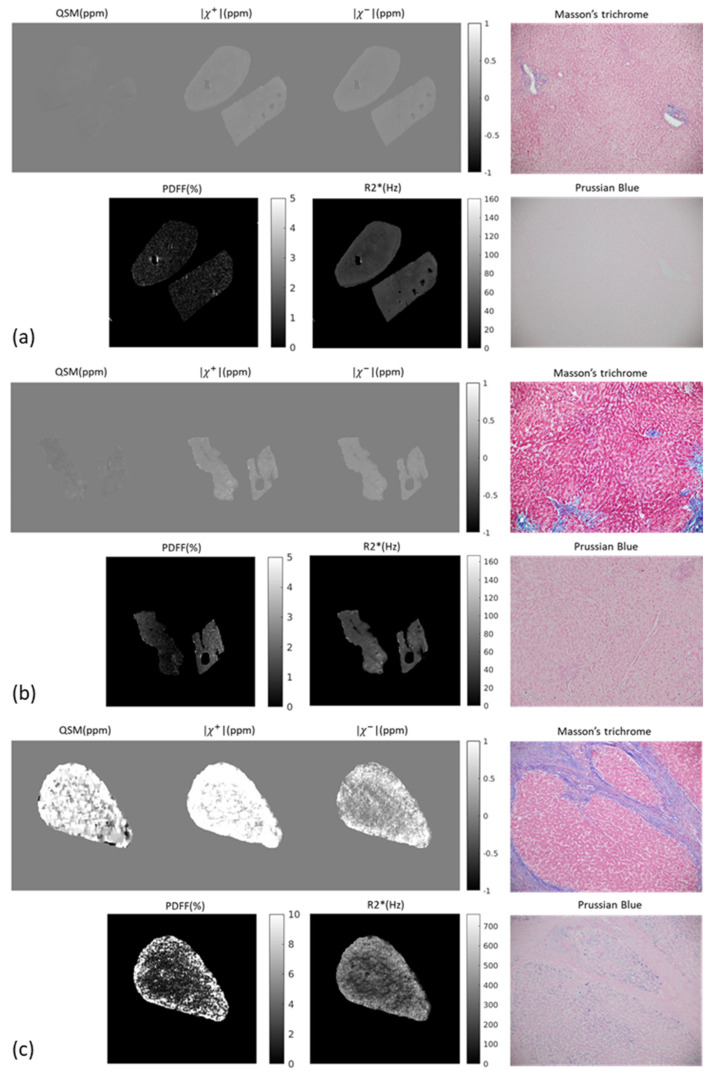
χ , $\left|\chi^{+}\right|$/$\left|\chi^{-}\right|$ maps (with optimized r obtained from leave-one-out cross-validation), R2* and PDFF in a cross-sectional slice through a nonfibrotic liver sample (**a**), intermediate-stage (F2) fibrosis case (**b**) and a cirrhotic liver sample (**c**). Masson’s trichrome and Prussian blue stains are shown for each example.

The average decay rate r value ± SD estimated for source separation using the leave-one-out technique was 144.2 ± 3.9 Hz/ppm (min: 140, max: 156), which differed substantially from the default value of 262 Hz/ppm [16,17]. Using this average decay rate as the optimized value, example images of $\left|\chi^{+}\right|$, $\left|\chi^{-}\right|$, as well as χ, R2* and PDFF maps are shown in Figure 1 along with pathologic stains.

**Figure 2:**
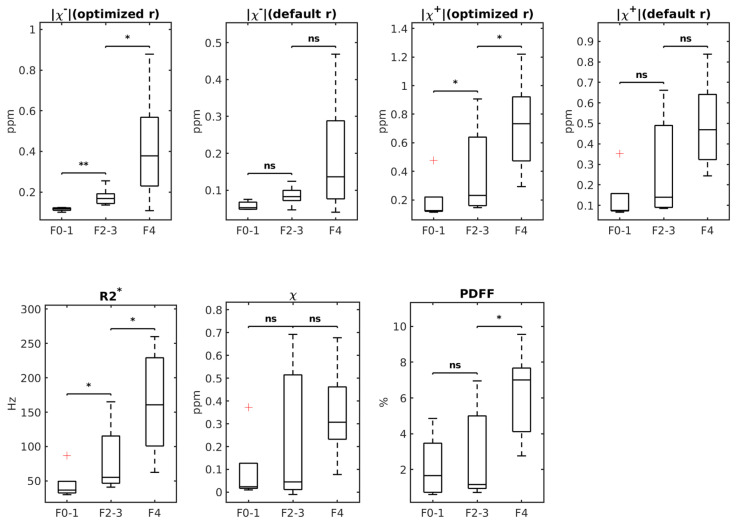
Boxplots of $\left|\chi^{-}\right|/\left|\chi^{+}\right|$ (with optimized r), $\left|\chi^{-}\right|/\left|\chi^{+}\right|$ (with default r), R2*, χ, and PDFF values in samples with stages F0–1, F2–3 and F4 respectively. The *p*-value range of a Mann–Whitney U test comparing the groups is displayed on top of the groups (ns: non-significant, *: 0.01 < *p* < 0.05, **: 0.001 < *p* < 0.01). The red plus signs stand for outliers in each group.

Figure 2 presents boxplots of $\left|\chi^{-}\right|$ calculated with optimized r obtained from leave-one-out cross-validation, $\left|\chi^{-}\right|$ calculated with default r, $\left|\chi^{+}\right|$ (optimized r), $\left|\chi^{+}\right|$ (default r), R2*, *χ* and PDFF across the subgroups of F0–1, F2–3 and F4 of liver fibrosis. When comparing both cirrhotic (F4) with medium-stage fibrosis (F2–3) and medium-stage fibrosis (F2–3) to none or mild fibrosis (F0–1), there were significant increases in $\left|\chi^{-}\right|$ with optimized r: F2–3 mean $\left|\chi^{-}\right|$ = 0.18±0.04 vs. F4 mean $\left|\chi^{-}\right|$ = 0.42±0.25 (*p* = 0.02) and F0–1 mean $\left|\chi^{-}\right|$ = 0.12± 0.01 vs. F2–3 mean $\left|\chi^{-}\right|$ = 0.18±0.04 (*p* = 0.0025). However, $\left|\chi^{-}\right|$ with default r showed no significant difference in both groups.

Similarly, when comparing cirrhotic (F4) with medium-stage fibrosis (F2–3), there were significant increases in the average R2* (p = 0.021), PDFF (*p* = 0.014) and $\left|\chi^{+}\right|$ with optimized r (*p* = 0.021) but not in $\left|\chi^{+}\right|$ with default r (*p* = 0.072); when comparing medium-stage fibrosis (F2–3) to none or mild fibrosis (F0–1), there was significant increase only in R2* (*p* = 0.048) and $\left|\chi^{+}\right|$ with optimized r (*p* = 0.048) but not in PDFF (*p* = 0.876) and $\left|\chi^{+}\right|$ with default *r* (*p* = 0.11). QSM total voxel susceptibility value χ was not statistically different across the groups (*p* > 0.05 for both comparisons), consistent with fibrosis and iron mixing across all stages.

**Figure 3:**
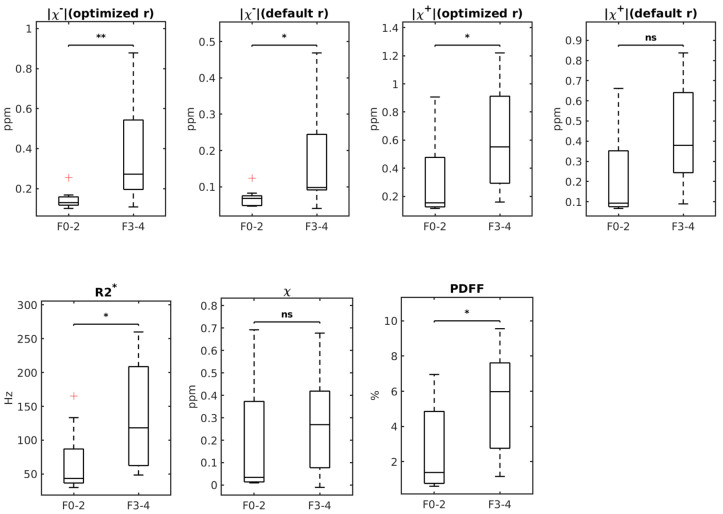
Boxplots of $\left|\chi^{-}\right|/\left|\chi^{+}\right|$ (optimized r), $\left|\chi^{-}\right|/\left|\chi^{+}\right|$ (default r), R2*, χ, and PDFF values in samples with stages F0–2 and F3–4. The *p*-value range of a Mann–Whitney U test comparing the groups is displayed on top of the groups (ns: non-significant, *: 0.01 < *p* < 0.05, **: 0.001 < *p* < 0.01). The red plus signs stand for outliers in each group.

Figure 3 shows boxplots of $\left|\chi^{-}\right|$ (optimized r), $\left|\chi^{-}\right|$ (default r), $\left|\chi^{+}\right|$ (optimized r), $\left|\chi^{+}\right|$ (default r), R2*, *χ* and PDFF across the subgroups of F0–2 and F3–4 of liver fibrosis. $\left|\chi^{-}\right|$ (optimized r), $\left|\chi^{-}\right|$ (default r), $\left|\chi^{+}\right|$ (optimized r), R2* and PDFF, not $\left|\chi^{+}\right|$ (default r) and χ all demonstrated significant differences between these two groups, with *p* = 0.0046, 0.026, 0.021, 0.014, 0.026, 0.054 and 0.27, respectively.

We found that positive susceptibility sources were able to distinguish between fibrosis groups. This result is expected, because iron—the liver’s primary paramagnetic content—can promote fibrosis when present in excess.

### 3.3. ROC Analysis for Differentiating Two Fibrosis Subgroups

**Figure 4:**
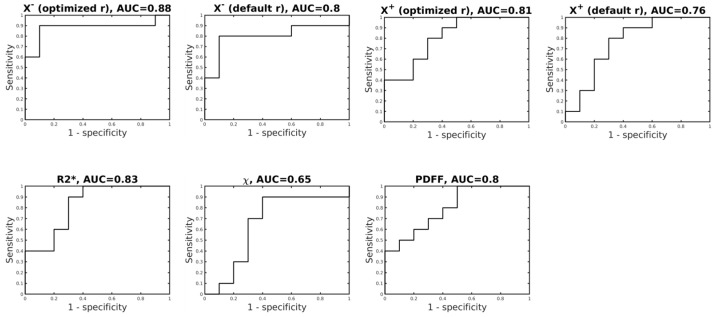
ROC curves for differentiation between samples in no fibrosis or with mild fibrosis F0–2 (n = 10) vs. samples with advanced fibrosis or cirrhosis F3–4 (n = 10) for $\left|\chi^{-}\right|/\left|\chi^{+}\right|$ (optimized r), $\left|\chi^{-}\right|$ (default r), R2*, χ, and PDFF.

Table 2 summarizes the ROC analysis results, including Area Under the Curve (AUC) and sensitivity and specificity, are detailed in Table 2, and Figure 4 displays Receiver Operating Characteristic (ROC) curves for the differentiation of fibrosis between subgroups of liver samples with stage F0–2 (n = 10) and stage F3–4 (n = 10). $\left|\chi^{-}\right|$ (optimized r) increased AUC to 0.88 (*p* = 0.0046), indicating a more accurate prediction than when R2* and QSM are used individually. In addition, R2*, $\left|\chi^{+}\right|$ (optimized r), $\left|\chi^{-}\right|$ (default r), and PDFF achieved AUCs = 0.83, 0.81, 0.8 and 0.8 and *p* = 0.014, 0.021, 0.026 and 0.026, respectively, for identifying significant-stage fibrosis/cirrhosis livers. $\left|\chi^{+}\right|$ (default r) and magnetic susceptibility (*χ*) did not reach statistical significance, with AUC = 0.76 and 0.65, and *p* = 0.053 and 0.27, respectively.

### 3.4. Correlation with Histology Grades

**Figure 5:**
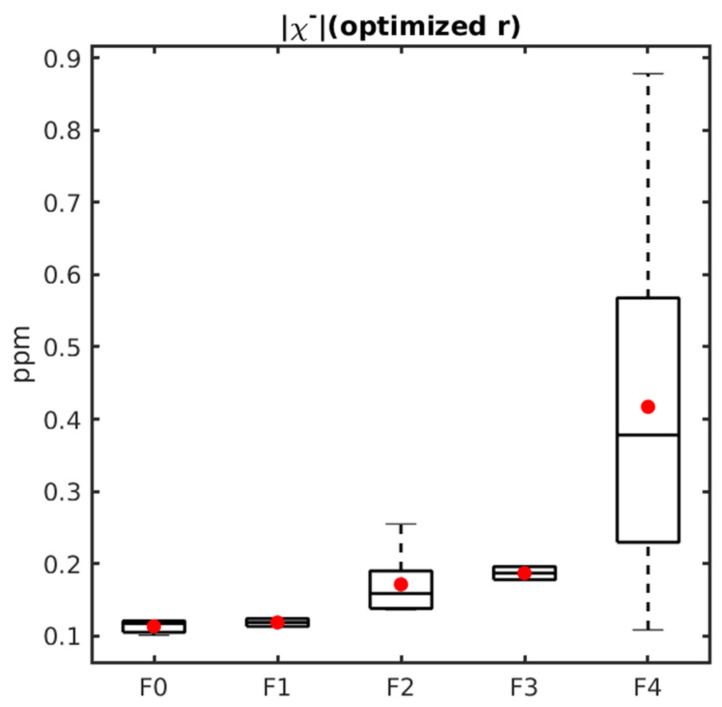
Boxplots of negative susceptibility sources with optimized r and their correlation with histology-derived fibrosis stage. The red dot for each category represents the average negative susceptibility sources of all the samples in the category.

Figure 5 shows the negative susceptibility sources obtained with source separation with optimized r. The measures correlate positively with the ordinal fibrosis stage determined from histology. For the $\left|\chi^{-}\right|$ with optimized r, the mean ± SD absolute negative susceptibility values were 0.11 ± 0.011, 0.12 ± 0.0077, 0.17 ± 0.049, 0.19 ± 0.013, and 0.42 ± 0.25 for samples classified as F0, F1, F2, F3, and F4, respectively. The Spearman correlation coefficients are r = 0.60.

### 3.5. $\chi^{+}$ Measurement for Iron

**Figure 6:**
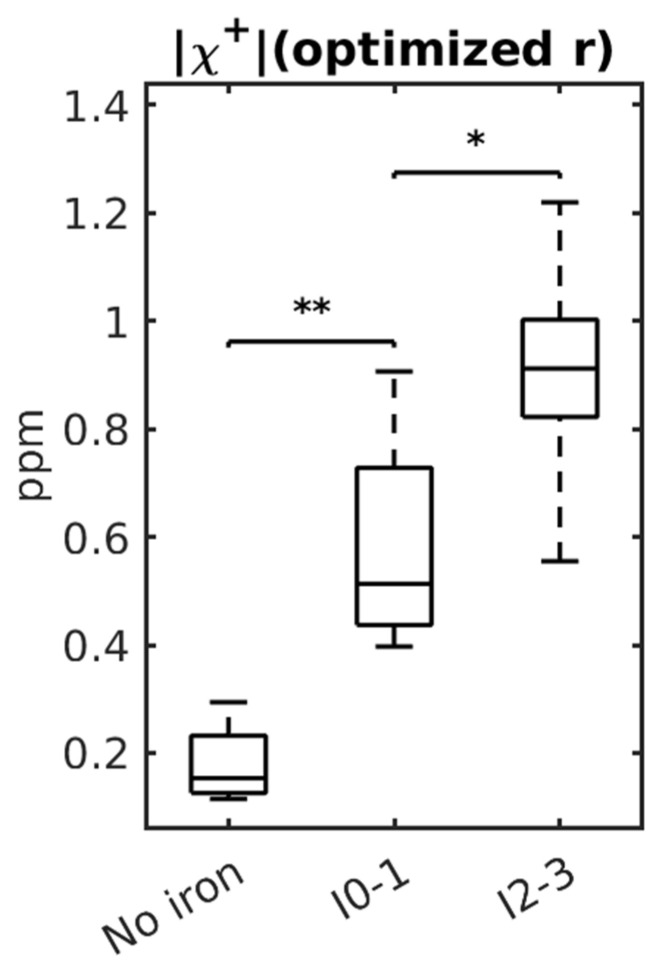
Boxplots of $\left|\chi^{+}\right|$ values with optimized r in samples with no iron, I0–1 and I2–3 iron, respectively. The *p*-value range of a Mann–Whitney U test comparing the groups is displayed on top of the groups (ns: non-significant, *: 0.01 < *p* < 0.05, **: 0.001 < *p* < 0.01).

Figure 6 shows iron content differed significantly between the non-iron and I0–1 groups (*p* = 0.002) and between the I0–1 and I2–3 groups (*p* = 0.032) for $\left|\chi^{+}\right|\;$with optimized r.

## 4. Discussion

Our results indicate that decay constant optimization for a specific tissue type is important in R2*QSM susceptibility source separation, and that diamagnetic susceptibility can serve as an accurate biomarker for assessing liver fibrosis: negative susceptibility differentiates fibrotic stages: none or mild fibrosis (F0–1), medium (F2–3), and cirrhotic (F4). Diamagnetic susceptibility is particularly useful in differentiating between livers with fibrosis F0–2 from those with >F2 as candidates for antifibrotic pharmacological treatment [31].

Liver susceptibility sources include diamagnetic fibrosis (prevalently collagen) and paramagnetic iron. In diseases such as nonalcoholic fatty liver disease (NAFLD) and steatohepatitis (NASH) (also together known as metabolic dysfunction-associated steatosis liver disease (MASLD)), fat is commonly present alongside liver fibrosis. To mitigate the impact of fat on QSM, we used water–fat separation to differentiate water and fat signals and adjust for fat content. To reduce the effect of paramagnetic sources like iron, we used susceptibility source separation to identify the diamagnetic susceptibility source for non-invasively assessing liver fibrosis using ex vivo liver explant samples. While QSM individually was not effective in identifying all groups of fibrosis stages, this study found that susceptibility source separation utilizing QSM and R2* from a single mGRE sequence is able to distinguish stage 0–1 from stage 2–3 (*p* = 0.0025) and stage 2–3 from cirrhotic/stage 4 (*p* = 0.021) in liver tissue, providing a ROC AUC of 0.88 for differentiating liver samples of stage F0–2 and advanced stage F3–4 fibrosis/cirrhosis.

Other relevant QSM methods include texture analysis using magnetic susceptibility and R2* to evaluate liver fibrosis, which has shown promising performance in differentiating fibrosis stages by quantifying spatial heterogeneity. Susceptibility tensor imaging (STI) has also been used to directly image diamagnetic susceptibility from collagen in hepatic fibrosis, suggesting a unique contrast mechanism to target fibrotic tissue components [32]. Source separation with optimized decay constant r demonstrated significantly better ability in differentiating both higher-stage fibrosis (F4 vs. F2–3 or F3–4 vs. F0–2) and lower-stage fibrosis F0–1 and F2–3 than source separation with default decay constant r.

Fibrosis is a very important concern in managing chronic liver disease (CLD), which has a molecular makeup of the fibrous scar tissue consisting of collagen types I and III, sulfated proteoglycans, and glycoproteins [33]. CLD worldwide affects 1.5 billion people, causing two million deaths each year [34,35]. The most common CLD etiologies are (i) NAFLD (or MASLD, as has been proposed [36], including nonalcoholic steatohepatitis, NASH, or metabolic dysfunction-associated steatohepatitis, MASH), (ii) viral infections (hepatitis B virus, HBV, and hepatitis C virus, HCV), and iii) alcohol-related liver disease (ALD); other etiologies include less common genetic, autoimmune, inflammatory, metabolic, infectious, and toxic disorders. In CLD, fibrosis, iron, and fat are interrelated and iron may be both a cause [37] and consequence of liver disease [38,39,40]. Fibrosis develops with excessive deposition of extracellular matrix macromolecules, predominantly collagens. The progressive formation of fibrous scars replaces normal tissue and distorts hepatic architecture. Cirrhosis develops following the formation of nodules of regenerating hepatocytes, potentially leading to portal hypertension, liver failure and hepatocellular carcinoma [41,42,43]. Noninvasive MRI is routinely used in managing patients with CLD, and liver fibrosis has been studied using R2 and R2* weighted imaging and magnetic resonance elastography (MRE). While MRE requires additional scanner hardware, R2 and R2* weighted methods have limited sensitivities; these methods may suffer from iron interference [44,45]. QSM from widely available gradient echo MRI data without any contrast injection or additional hardware is well suited for longitudinal quantitative monitoring [46]. The R2*QSM source separation-based method presented here offers a promising, widely accessible method for studying liver fibrosis.

This study has several limitations. First, only 20 samples were included in the final analysis, with an uneven distribution across fibrosis stages, which may limit statistical robustness. To validate these findings and assess their applicability in clinical environments, further studies are necessary, involving larger participant groups. Second, the source separation physical model involves substantial simplification and requires further investigation. Third, histological staging was derived from localized tissue sections, whereas MRI-derived susceptibility metrics were averaged over substantially larger specimen volumes. Given the spatial heterogeneity of fibrosis, this scale mismatch may introduce bias in the statistical analyses by underrepresenting focal fibrotic regions. Fourth, previous phantom studies demonstrate the strong nonlinear effects of fibrosis and fat interference on the R2* estimate [47] and of signal dependence on voxel size [48]. In the decay constant formulation, Equation (3) is only approximate. Therefore, residual iron effects may therefore influence the diamagnetic susceptibility estimates, especially in F4 samples, and future work should introduce more refined biophysical models that include cross terms between positive and negative susceptibility sources. Also, more accurate numerical formulation may be used to model susceptibility sources including deoxyheme iron related to hepatocyte oxygen metabolism and ferritin iron with a unique magnetization saturation property [49,50,51]. In addition, formalin fixation may cause an R2* increase [52,53], and may have affected the evaluation of susceptibility sources and other MRI parameters in our study. Results here warrant future study with fresh liver samples without formalin fixation, and the reproducibility of the results on different scanners and in vivo using the decay constant values so determined should be further studied. Additionally, although large vessels were excluded when drawing the masks, segmentation inaccuracies are still likely in regions near vessels. Finally, the decay constant r was optimized using fibrosis-stage discrimination as the objective function. While this approach improves performance within the current dataset, the optimal value of r may depend on tissue properties, voxel size, disease etiology, and acquisition conditions, and therefore may not be universally transferable. From a practical standpoint, the current optimization and reconstruction pipeline was implemented in MATLAB and involves multiple processing steps, which may limit immediate clinical deployment. Translation to routine clinical use will require streamlined, automated implementations integrated into scanner workflows or clinical image-processing platforms, as well as a validation of computational efficiency and robustness.

In future in vivo or multicenter studies, r could be calibrated using a training cohort and then fixed for independent testing, or alternatively modeled as a function of measurable imaging parameters such as field strength, voxel size, echo spacing or repetition time to improve robustness across scanners. As a result, optimal cut-off can be recalculated once the retraining is performed. Second, the proposed method against established noninvasive fibrosis assessment techniques such as magnetic resonance elastography (MRE) or diffusion-weighted imaging (DWI) can be studied on the same dataset. Finally, another direction that could be explored in the future is conducting longitudinal in vivo studies to determine whether the proposed susceptibility-based biomarkers can support disease monitoring.

## 5. Conclusions

In conclusion, our study shows that the diamagnetic susceptibility source obtained from mGRE data alone is a promising tool for the noninvasive diagnosis of the liver fibrosis stage, which may be used as an alternative to liver biopsy.

## Figures and Tables

**Table 1:** Demographic and histopathologic characteristics of the liver samples.

	All (n = 20)	F0–1 (n = 5)	F2–3 (n = 7)	F4 (n = 8)
Age (y)	45 (0–80)	26 (0–80)	45 (24–61)	57 (49–66)
Sex				
-Male	13	4	4	5
-Female	7	1	3	3
Iron deposition				
-None	10	4	5	1
-Grade 0	1	0	1 *	0
-Grade 0–1	1	0	1	0
-Grade 1	1	1	0	2
-Grade 2	1	0	0	1
-Grade 2–3	3	0	0	3
-Grade 3	1	0	0	1
Steatosis				
-None	9	2	6	1
-Grade 0 (<5%)	5	1	0	4
-Grade 1 (5–33%)	4	1	1	2
-Grade 2 (34–66%)	1	1	0	0
-Grade 3 (>66%)	1	0	0	1

* In one sample where grade 0 iron was found in the hepatocytes, iron was observed in the Kupffer cells.

**Table 2:** Results of ROC analysis of MRI parameters for differentiation between liver samples with lower-stage fibrosis (F0–2, n = 10) and advanced liver fibrosis/cirrhosis (F3–4, n = 10).

Parameters	AUC	*p*-Value	Cut-Off	Sensitivity (%)	Specificity (%)
$\left|\chi^{-}\right|$ (optimized r) (ppm)	0.88	0.0046	0.17	90	90
R2* (s^−1^)	0.83	0.014	87.02	90	70
$\left|\chi^{+}\right|$ (optimized r) (ppm)	0.81	0.021	0.39	80	70
$\left|\chi^{-}\right|$ (default r) (ppm)	0.80	0.026	0.074	90	80
PDFF (%)	0.80	0.026	6.79	100	50
$\left|\chi^{+}\right|$ (default r) (ppm)	0.76	0.053	0.27	80	70
χ (ppm)	0.65	0.27	0.29	90	60

## Data Availability

The data presented in this study are available on reasonable request from the corresponding author.
